# 374. Early Discontinuation of Empiric Antibiotics for Febrile Neutropenia in Pediatric Allogeneic Haploidentical Hematopoietic Cell Transplant Recipients

**DOI:** 10.1093/ofid/ofae631.115

**Published:** 2025-01-29

**Authors:** Sandra Castejon Ramirez, Jose Amadeo A Ferrolino, Kim J Allison, Pamela Merritt, Megan Peterson, Amanda Cole, Amber Davis, Ashleigh Gowen, Li Tang, Guangjin Luo, Ronald H Dallas, Rachel L Wattier, Diego R Hijano, Gabriela Maron

**Affiliations:** St. Jude Children's Research Hospital, Memphis, TN; St. Jude Children's Research Hospital, Memphis, TN; St. Jude Children's Research Hospital, Memphis, TN; St. Jude Children's Research Hospital, Memphis, TN; St. Jude Children's Research Hospital, Memphis, TN; St. Jude Children's Research Hospital, Memphis, TN; St. Jude Children's Research Hospital, Memphis, TN; St. Jude Children's Research Hospital, Memphis, TN; St. Jude Children's Research Hospital, Memphis, TN; St. Jude Children's Research Hospital, Memphis, TN; St. Jude Children's Research Hospital, Memphis, TN; University of California, San Francisco, San Francisco, CA; St. Jude Children's Research Hospital, Memphis, TN; St. Jude Children's Research Hospital, Memphis, TN

## Abstract

**Background:**

Febrile neutropenia (FN) occurs in 80-90% of haploidentical hematopoietic cell transplants (haplo-HCT), but most patients do not have documented infections. Empiric antibiotics are frequently continued until neutrophil recovery, even when there is no evidence of infection. Shorter antibiotic courses to decrease antibiotic exposure have been studied in adult HCT recipients and have demonstrated safety compared to conventional management. In 2017 we implemented a strategy to discontinue antibiotics at ≤ 72 h in stable haplo-HCT patients with FN and no evidence of infection. In this study, we evaluate the implementation and outcomes of this practice.

**Figure 1.**
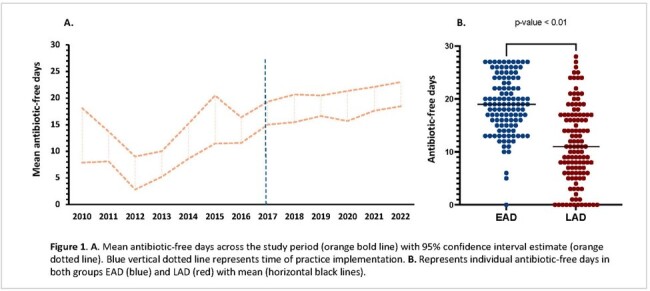
A. Mean antibiotic-free days across the study period. B. Individual antibiotic-free days in EAD and LAD

**Methods:**

This single-center retrospective cohort included patients < 24 years old who underwent haplo-HCT from 2010 to 2022 with ≥ 1 episode of FN and no documented infection. They were classified by time period as follows: late antibiotic discontinuation (LAD) vs. early antibiotic discontinuation < 72 h (EAD). The primary outcome was antibiotic-free days. The secondary outcomes were subsequent breakthrough bloodstream infection (BSI), intensive care unit (ICU) admission, septic shock, mortality, and practice fidelity (adherence) and sustainability.

**Figure 2.**
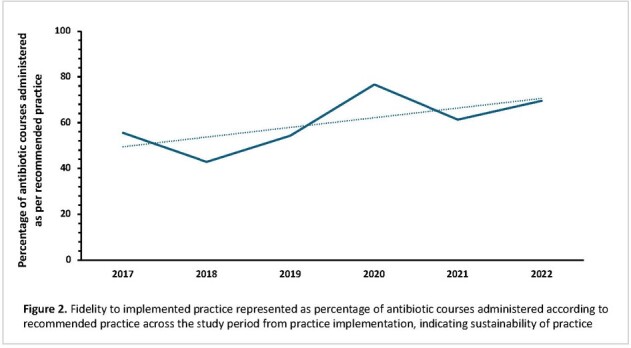
Fidelity to implemented practice across time

**Results:**

We included 230 patients (117 in LAD, 113 in EAD). No differences in age, sex, or race were observed. A higher proportion of patients received myeloablative conditioning regimens in LAD (p < 0.01, Table 1). There were more antibiotic-free days after EAD implementation (Figure 1), but no differences in the incidence of breakthrough bloodstream infections (EAD: 6.1%, LAD: 5.1%, p= 0.81) or other adverse clinical outcomes. There was no infection-related mortality in either group (Table 1). The fidelity (adherence) to recommended practice of discontinuing antibiotics ≤72 hours was 61.6%, with increase over time (Figure 2).

**Figure:**
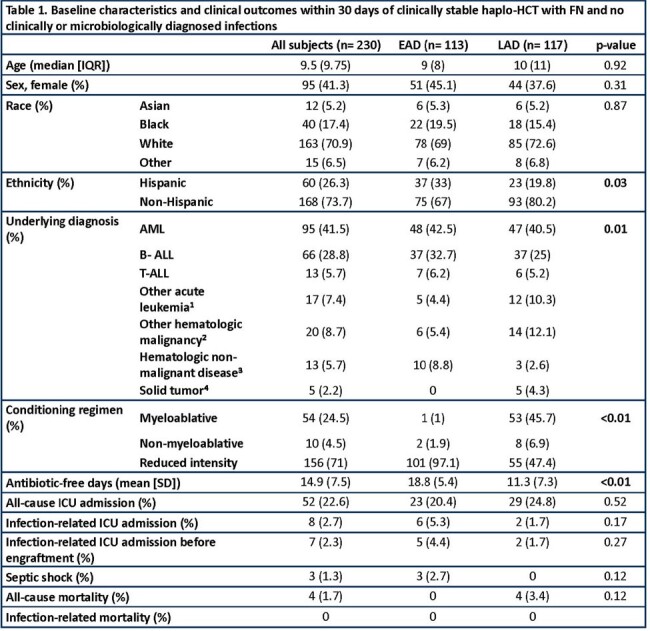

**Conclusion:**

Implementation of EAD strategy for FN in haplo-HCT patients led to an increase in antibiotic-free days during the first 30 days post-HCT. Adverse clinical outcomes were infrequent in both groups and did not significantly increase after EAD. Prospective multicenter studies are required to evaluate the safety of EAD strategies.

**Disclosures:**

**Diego R. Hijano, MD, MSc**, FDA: Grant/Research Support|Merck: Grant/Research Support|National Institute of Health: Grant/Research Support **Gabriela Maron, MD, MS**, Astellas Inc: Grant/Research Support|NIH: Grant/Research Support|SymBio Pharma: Advisor/Consultant|SymBio Pharma: Grant/Research Support

